# The Sublethal Effects of Neonicotinoids on Honeybees

**DOI:** 10.3390/biology14081076

**Published:** 2025-08-18

**Authors:** Zunair Ahsan, Zhijia Wu, Zheguang Lin, Ting Ji, Kang Wang

**Affiliations:** College of Animal Science and Technology, Yangzhou University, 88 South University Rd, Yangzhou 225009, China; mh24057@stu.yzu.edu.cn (Z.A.);

**Keywords:** neonicotinoids, honeybees, sublethal effects, neurotoxicity, colony collapse, behavioral impairment

## Abstract

Honeybees (*Apis mellifera*) are indispensable pollinators vital to global biodiversity, ecosystem stability, and agricultural productivity, and they promote over 35% of food crops and 75% of flowering plants. Yet, they are in unprecedented decline, partly as a result of neonicotinoid pesticide use elsewhere. These effects on honey bee health are synthesized in this paper through molecular, physiological, and behavioral data showing that sublethal effects of neonicotinoids impair honey bee health. As neurotoxic insecticides that target nicotinic acetylcholine receptors (nAChRs), these insecticides interfere with neurotransmission and underlie cognitive impairment, immune suppression, and oxidative stress. Developmental toxicity is manifested in larvae as retarded growth, reduced feeding, and increased death; queen and drone reproduction are impaired, lowering colony viability. As a result, adult bees have shortened lives and erratic foraging, are further disoriented, and experience impaired navigation, communication, and resource collection. Together, these effects cascade to reduced brood care, thermoregulatory failure, and heretofore unrecognized increased susceptibility to pathogens, increasing the probability of colony collapse at the colony level. Contaminants such as pesticides may cause pollinator exposure and, in turn, may cause their population to be undermined if they are not mitigated; therefore, urgent mitigation strategies, including integrated pest management (IPM), regulatory reforms, and adoption of biopesticides, are needed to mitigate pollinator exposure. The focus of this review lies in the ecological necessity of restructuring how agriculture is managed to simultaneously meet food security and the conservation of honeybee health, the linchpin of global ecosystems.

## Simple Summary

Honeybees, crucial for pollinating our food crops and wildflowers, are facing a serious decline. A major reason for this is the use of certain pesticides called neonicotinoids. This paper shows how even small amounts of these pesticides harm bees by disrupting their brain function, weakening their immune system, and causing stress. Young bees grow poorly and die more often, while adult bees have shorter lives and struggle to find food and navigate. This leads to problems within the hive, like poor brood care and increased vulnerability to diseases, ultimately making colonies more likely to collapse. Since these pesticides are a significant threat to pollinators, this paper emphasizes the urgent need for new ways to manage agriculture. The solution includes using methods that reduce pesticide use, changing regulations, and adopting safer alternatives. By protecting honeybee health, we can ensure both our food supply and the health of the entire planet.

## 1. Introduction and Definitions

More than 75% of flowering plants and 35% of crop production are reproduced by pollinators, especially honeybees (*Apis mellifera*), which are essential to agriculture and global ecosystems. However, habitat loss, pathogens, climate change, and pesticide exposure are all contributing to the global decline in pollinator populations [1]. Neonicotinoids are a class of systemic insecticides that have caused a great deal of concern among pesticide users because of their extensive use, environmental persistence, and sublethal effects on bees [2]. Even at sublethal dosages, neonicotinoids, like imidacloprid and clothianidin, are neurotoxicants affecting honeybee immune function, learning, navigation, and foraging behavior. Long-term population declines, increased vulnerability to parasites, and decreased colony fitness have all been associated with chronic exposure, especially close to agricultural landscapes [3]. Understanding the sublethal effects of neonicotinoids is essential for creating sustainable pest management plans and reducing pollinator losses, especially considering the ecological and economic significance of honey bees [4]. Nicotinic acetylcholine receptors (nAChRs) in the insect nervous system are the specific target of neonicotinoids, a class of systemic insecticides that are chemically modeled after nicotine [5]. Because of their great effectiveness against pest insects that feed on sap and chew [6], these insecticides have become the most commonly used class of insecticides worldwide. Although they still have notable sublethal effects on non-target organisms like pollinators, their selective toxicity results from a higher binding affinity to insect nAChRs than vertebrate receptors [5]. Neonicotinoids’ systemic nature is one of their distinguishing features; they are taken up by plants and transported to all plant parts, including pollen and nectar, via vascular tissues, rendering the entire plant poisonous to insects that feed on it [7]. Imidacloprid is the first commercially successful neonicotinoid that is still widely used despite growing regulatory restrictions. Other commonly used neonicotinoid compounds include acetamiprid, clothianidin, thiamethoxam, imidacloprid, and thiacloprid. Chronic environmental exposure results from these pesticides’ remarkable environmental persistence, which keeps them active in soils and water for months to years [6]. While dust produced during the planting of treated seeds represents another important pathway of environmental dispersion and non-target exposure, their high water solubility aids in leaching into waterways [7]. Neonicotinoids are especially dangerous to non-target organisms, such as pollinators, that come into contact with the insecticides through a variety of exposure pathways, systemic action, and environmental persistence [4]. Determining acute lethal effects using standardized metrics like LD50 or LC50 values over brief exposure periods has been the main focus of traditional toxicological testing of pesticides [8]. Under controlled conditions, these laboratory-based assays usually assess mortality in individual organisms, but they frequently fall short in simulating the complexity of the environment in the real world [9]. The focus on individual-level responses rather than population- or colony-level impacts is a major drawback of traditional testing procedures [10]. Essential ecological elements like social interactions, foraging dynamics, and cumulative exposure to multiple stressors are often overlooked in standard laboratory tests for honey bees [9]. Furthermore, because current methodologies fail to adequately address chronic exposure effects that may manifest at low doses over extended periods of time, they frequently underestimate risks. Sublethal effects are frequently subtle and may only manifest after extended or repeated exposure, in contrast to acute toxicity, which can be readily measured using mortality assays [11]. Neonicotinoids, for instance, can interfere with honey bees’ ability to dominate at sublethal levels, increasing forager loss and decreasing colony productivity [12]. In a similar vein, long-term exposure may affect social communication, queen reproduction, and brood care, all of which are essential for colony sustainability [13]. This emphasizes the necessity for regulatory frameworks to include sublethal endpoints in risk assessments and to go beyond conventional lethality testing [8]. By examining three crucial elements identified in recent research, this review investigates the sublethal effects of neonicotinoid insecticides on honey bees (*Apis mellifera*).

## 2. General Biochemistry and Physiology

### 2.1. Overview of Neonicotinoids

Chemically related to nicotine, neonicotinoids are a common type of systemic insecticides that are highly effective against insect pests and have comparatively little toxicity to vertebrates. Because of their systemic qualities, great efficacy against a wide variety of pests, and adaptability, they have become the most widely used insecticides in the world, making up over 25% of the market [14]. Neonicotinoids are structurally composed of a heterocyclic core, usually a thiazolyl methyl or chloropyridinyl moiety connected to an electronegative group such as nitro or cyano function, which is essential for binding to the insect nicotinic acetylcholine receptor (nAChR) [15]. While showing far less activity at mammalian nAChRs, these substances function as agonists at insect nAChRs, resulting in prolonged neuronal excitement, paralysis, and death [16]. Because of their advantageous physicochemical properties, which include high water solubility and chemical stability, neonicotinoids may be efficiently delivered to insect feeding areas and absorbed systemically by plants [17]. Because of their tenacity and environmental mobility, they have been found in sediments, surface water, and even sources of drinking water, which raises questions about the effects on the environment and human health [18]. Imidacloprid, clothianidin, thiamethoxam, acetamiprid, thiacloprid, and dinotefuran are common neonicotinoids that have been found in urban and agricultural environments worldwide [19]. Neonicotinoids were first created as safer substitutes for organophosphates and carbamates, but new research has revealed that their extensive use endangers ecosystems and non-target species, particularly pollinators and aquatic invertebrates [20]. These difficulties are forcing a review of regulations and a hunt for better or substitute pesticides with less of an adverse effect on the environment [21].

### 2.2. Structure and Receptor Selectivity of Neonicotinoids

Neonicotinoids are structurally similar in that they have a heterocyclic ring with an electron-withdrawing nitro or cyano group that increases their binding affinity to insect nAChRs [22]. These substances are particularly effective against pests that feed on sap because they are extremely water soluble and systemic, allowing for uptake and movement within plants [16]. The cholinergic system in insects is essential for synaptic communication, regulating processes like movement, memory, and learning [23]. The principal excitatory neurotransmitter in the central nervous system of insects is acetylcholine (ACh), which binds to two different types of receptors: nicotinic AChRs (nAChRs), which are ion channels and the main targets of neonicotinoids, and muscarinic AChRs (mAChRs), which are G-protein coupled [24]. A wide variety of nAChR subunits are found in insects, such as α1–α9 and β1–β2 in honey bees, which can form homomeric or heteromeric pentameric receptors [25]. These subunits’ precise makeup directly affects sensitivity to neonicotinoids; particular subunit combinations raise the binding affinity for pesticides such as clothianidin and imidacloprid [26]. Although recent research indicates that neonicotinoids may have effects on mammalian receptors at high concentrations, they notably bind to insect nAChRs more preferentially than their vertebrate counterparts, providing selective toxicity [16]. Certain insect nAChRs, especially those with particular α subunits, are also sensitive to α-bungarotoxin, which may have an impact on receptor pharmacology and neonicotinoid sensitivity [25]. Lastly, newer substitutes for neonicotinoids, like butenolides (like flupyradifurone) and sulfoximines (like sulfoxaflor), target related receptor subtypes while attempting to lower hazards to non-target organisms [27].

#### 2.2.1. Neural Targets and Functional Disruption

Nicotinic acetylcholine receptors (nAChRs) are found in major neuronal structures like the central complex (CC), antennal lobes (ALs), and mushroom bodies (MBs) in insect brains. These receptors mediate vital functions like memory, learning, and sensory integration [28]. Both the anatomical regions of expression and the orthosteric binding domains on receptor proteins where neonicotinoids act are referred to as these receptor “sites”. In order to cause overstimulation and neuronal dysfunction, neonicotinoids mostly bind to the orthosteric (agonist) regions of insect nAChRs [15]. The subunit composition of nAChRs, which comprises a variety of α and β subunits (such as α1–α9 and β1–β2 in honey bees), is crucial in determining the toxicity and binding affinity of different neonicotinoids [29]. Neonicotinoids’ selective toxicity to insects is caused by their high-affinity binding to particular nAChR subunit interfaces, such as α–β and α–α, according to structural studies [22]. 

#### 2.2.2. Receptor Subtypes and Molecular Mechanisms

Nicotinic acetylcholine receptors (nAChRs) in insects, such as honey bees, are made up of different combinations of α and β subunits, resulting in several functional subtypes, including α1/β1, α8/β1, and α1/α8/β1 [30]. Different pharmacological sensitivity to neonicotinoid pesticides is conferred by these subunit combinations. For instance, thiacloprid and clothianidin may interact differentially according on the subunit makeup, but imidacloprid exhibits a strong affinity for α1/β1-type receptors [31,32]. Interestingly, insects’ α-bungarotoxin (α-Bgt)-sensitive nAChRs match receptors with α7-like subunits, which reflect high-affinity neonicotinoid binding sites and structurally resemble vertebrate α7 [33]. The principal excitatory neurotransmission system in insects, the cholinergic system controls vital functions like motor control, memory and learning, olfactory processing, sensory integration, and spatial navigation [24]. Cholinergic neurons and nicotinic acetylcholine receptors (nAChRs) are abundantly expressed in three important brain regions in honey bees: the central complex (CC), which mediates motor control and navigation; antennal lobes (ALs), which process smells; and mushroom bodies (MBs), which are involved in memory and learning [34]. Normal cholinergic signaling in insect nAChRs is disrupted by neonicotinoids, which function as agonists at these receptors. Reduced learning capacity, impaired memory formation, disturbed olfactory processing, and navigational errors that show up behaviorally as confused foraging, an inability to do the waggle dance, and decreased colony survival are the outcomes of this [35].

In addition to targeting cholinergic signaling, neonicotinoids disrupt several non-cholinergic neurotransmitter systems in insects, including dopaminergic, serotonergic, glutamatergic, and GABAergic pathways. Overactivation of presynaptic nAChRs by compounds such as thiacloprid and clothianidin leads to excessive neuronal depolarization and dysregulated neurotransmitter release. This persistent stimulation alters dopamine and serotonin signaling, which are critical for motor coordination, reward-based learning, and motivational behaviors. For example, neonicotinoid exposure has been shown to increase dopamine release by activating α4β2 and α7 nAChR subtypes in the insect brain, while simultaneously impairing serotonin-mediated motor control, as demonstrated in *C. elegans* models where serotonin supplementation partially rescued locomotor deficits [36]. Furthermore, neonicotinoids reduce expression of vesicular glutamate transporters (VGLUTs), leading to weakened glutamatergic transmission in central circuits and at neuromuscular junctions—disrupting sensory processing and motor output [16]. Chronic nAChR stimulation also results in abnormal calcium influx, which suppresses GABAergic inhibitory signaling and induces hyperexcitability in the nervous system. This excitatory/inhibitory imbalance is associated with seizure-like neural activity and a breakdown of coordinated behaviors [37]. Additionally, prolonged calcium dysregulation leads to oxidative stress and mitochondrial dysfunction, contributing to long-term neurotoxicity [38]. Structural impairments have also been reported; for instance, reduced synaptic density in the mushroom bodies and antennal lobes of Drosophila has been linked to deficits in memory and olfactory processing [35]. In insects, glutamate plays a crucial role as a major excitatory neurotransmitter, functioning not only within the central nervous system (CNS) but also at the neuromuscular junction (NMJ). This is in contrast to vertebrates, where glutamate typically functions primarily within the CNS. At the insect NMJ, glutamate facilitates motor control and synaptic plasticity, making it essential for coordinated movement and behavioral responses [39]. Additionally, the Drosophila NMJ has been a valuable model for understanding glutamatergic synapse function, with studies showing that impaired glutamatergic signaling leads to locomotion defects and motor dysfunction [40]. Glutamate also contributes to sensory processing and neural plasticity, particularly in circuits involved in visual and mechanosensory input. Vesicular glutamate transporters (VGLUTs) are essential for loading glutamate into synaptic vesicles. When VGLUT expression is downregulated, synaptic excitation weakens due to impaired glutamate release, leading to diminished motor ability and behavioral performance deficits [41]. Neonicotinoid exposure, such as from imidacloprid, has been shown to impair glutamatergic synaptic plasticity in Drosophila. This results in desensitized behavioral responses and reduced short-term synaptic facilitation at NMJs, further emphasizing the role of glutamate in maintaining effective excitatory signaling [42]. α-Bungarotoxin (α-Bgt)-sensitive receptors primarily refer to specific subtypes of nicotinic acetylcholine receptors (nAChRs), notably the homomeric α7 and muscle-type nAChRs. These receptors exhibit high binding affinity to α-Bgt, distinguishing them from other subtypes like α4β2, which show negligible binding. Recent studies emphasize the complexity and diversity within nAChR subtypes. For instance, the discovery of heteromeric α7β2 nAChRs has revealed a subtype with potentially unique functional roles, particularly in neurodegeneration, although it appears to have reduced affinity for α-Bgt, suggesting altered structural binding sites [43]. Additionally, synthetic peptides that mimic the C-loop of α1 and α7 subunits have been shown to bind α-Bgt with nanomolar affinity but not similar toxins like α-cobratoxin, highlighting the receptor’s strict subtype specificity [44]. In the context of receptor biology, it is essential to distinguish between binding specificity and receptor affinity. Binding specificity refers to the selective recognition of a receptor subtype by a ligand, while receptor affinity quantifies the strength of that interaction. These properties do not always align; a ligand may bind strongly (high affinity) but to multiple receptor subtypes (low specificity). This distinction was clearly demonstrated in a study on α1A-adrenergic receptors, where certain compounds showed strong affinity but low specificity, and vice versa [45]. Furthermore, computational tools like AlphaFold have been applied to predict peptide-receptor binding affinities, offering insights into how structural complementarity drives high-affinity interactions independent of specificity [46]. The central complex (CC), a key brain structure in insects, is involved in sensory integration and motor control. Though direct data on α-Bgt-sensitive receptors in the CC is limited, this region is known to express cholinergic systems and likely harbors diverse nAChRs, including potentially α7-containing subtypes relevant for cholinergic signaling. The diversity of nAChR subtypes, formed from various combinations of α (α2–α10) and β (β2–β4) subunits, underpins their functional specialization. A recent computational modeling study demonstrated the successful engineering of α-conotoxins to selectively bind specific nAChR subtypes, illustrating the therapeutic potential of targeting receptor subtype diversity [47]. Through transcriptomic, proteomic, and electrophysiological research, the molecular mechanisms behind their toxicity in honey bees are being better understood, despite their high selectivity for insects over vertebrates [48].

### 2.3. Disruption of Neurotransmission and Signaling Pathway

Neonicotinoids disrupt key signaling molecules involved in synaptic function by affecting multiple neurotransmitter systems beyond the cholinergic pathway. These pesticides cause excitatory circuits to become overactive and inhibitory signals to be suppressed by disrupting the balance between excitatory and inhibitory neurotransmission. Research has demonstrated that long-term exposure to neonicotinoids changes dopamine and serotonin signaling, impairing honeybees’ cognitive and motor abilities. Neuronal dysfunction is further aggravated by neonicotinoid-induced disruptions in the phosphoinositide 3-kinase (PI3K) and mitogen-activated protein kinase (MAPK) pathways. After being exposed to neonicotinoids, the MAPK pathway, which is essential for neuronal survival and plasticity, it has been demonstrated to decrease the expression of vesicular glutamate transporters (VGLUTs), which are critical for maintaining for preserving excitatory neurotransmission with long term exposure. Glutamatergic signaling is diminished as a result of this downregulation, which impacts neuronal communication and synaptic effectiveness. A further factor in synaptic failure is inhibition of voltage-gated calcium channels (VGCCs), which impairs motor coordination and sensory processing [49]. Cytochrome P450 monooxygenases (P450s), a superfamily of enzymes that metabolize xenobiotic substances, including insecticides, are largely responsible for detoxifying neonicotinoid pesticides in honey bees. These enzymes affect honey bees’ resistance and susceptibility to chemical exposures and are important in the metabolism of pesticides. However, honey bees have a relatively limited detoxification capacity and are therefore more susceptible to toxic compounds because their genomes contain fewer *P450* genes than those of other insect species [50]. The comparatively lower toxicity of thiacloprid to honey bees in comparison to imidacloprid can be explained by this differential enzymatic activity. According to expression analyses, worker bee brains and Malpighian tubules tissues vital to detoxification and neurological processes exhibit high *CYP9Q3* levels, indicating its strategic role in mitigating insecticide toxicity [51,52]. The detoxification process is intricate, though, and the metabolic intermediates that *P450* activity produces can occasionally be more harmful than the parent substances, putting the bees at further risk [53]. The impact of the honey bee gut microbiota, which has been demonstrated to significantly regulate the expression of *P450* genes in the digestive tract, further complicates the effectiveness of detoxification. In particular, glyphosate-based pesticides have been shown to significantly change the gut microbial community’s composition, subsequently reducing detoxification ability and increasing honey bee mortality [54]. Although *P450* enzymes are the mainstay of honey bee pesticide detoxification, both extrinsic (such as exposure to environmental pesticides and the makeup of the gut microbiota) and intrinsic (such as tissue-specific expression and enzyme specificity) factors influence how effective these enzymes are *Apis mellifera*’s increased susceptibility to due to their limited diversity and number of *P450* genes relative to other insects [52].

### 2.4. Environmental Influences on Gene Expression

The ability of honey bees to metabolize toxins is improved by dietary phytochemicals, particularly p-coumaric acid from pollen, which significantly upregulates multiple cytochrome P450 (P450) genes, including six genes from the CYP6AS subfamily and one from the CYP9Q subfamily [55]. Furthermore, bees with a conventional gut microbiota (CV) have significantly higher midgut expression of P450 genes, such as CYP6AS1, CYP6AS3, CYP6AS4, CYP6AS10, CYP9Q2, and CYP9Q3, than bees with a disrupted or deficient gut microbiota (GD). This suggests that the composition of the microbiome directly controls genetic expression linked to detoxification [56]. Additionally, pollen nutrition influences honey bees’ ability to withstand pesticides; high-quality pollen diets (S pollen) increase the bees’ resistance to azoxystrobin and sulfoxaflor. Additionally, bees fed the high-quality pollen diet exhibit faster pesticide metabolization and higher levels of vitellogenin, a glycoprotein linked to longevity and resistance to oxidative stress [57]. But when sublethal doses of neonicotinoids are combined with stressors like *Nosema ceranae* infection, detoxification genes are expressed differently, which reduces the efficiency of pesticide metabolism [48]. Although detoxification procedures enable honey bees to withstand certain xenobiotics, they are energy-intensive and put additional metabolic strain on the body, which may result in trade-offs with other physiological functions [58]. Furthermore, metabolized neonicotinoids, which can occasionally become even more toxic than their parent compounds, can worsen the detrimental effects on bees [53].

### 2.5. Caste-Based Differences in Susceptibility Within Apis mellifera

Due to variations in physiology, detoxification processes, behavior, and genetics, honeybees of different castes (workers, queens, and drones) and species are more or less susceptible to neonicotinoids. Because they forage, worker bees are particularly susceptible to direct exposure to pollen, contaminated nectar, and flowers treated with pesticides [59]. Despite being less directly exposed, queens may suffer indirect consequences from workers’ tainted food, which could harm their reproductive health [60]. Because they are exposed to a lot of foraging, worker bees have been found to have the highest mortality rates because worker bees are more vulnerable than queens because they come into contact with neonicotinoid-contaminated resources more frequently [59]. Workers experience cumulative toxicity as a result of extended exposure, even though their detoxification system is more active. Queens exhibited sublethal effects like decreased egg-laying capacity and weakened colony stability, despite having lower direct exposure [60]. Neonicotinoid exposure interferes with normal physiological and behavioral processes, even though they did not go into great detail about behavioral or physiological adaptations to imidacloprid exposure. The development and health of colonies can be hampered by the decreased locomotor activity and egg-laying of queens, as well as the decreased foraging and hygienic practices of workers [61]. Furthermore, in order to reduce pesticide exposure, *Apis mellifera* has evolved adaptive responses like modified foraging habits and social interaction modifications; however, these adaptations are frequently insufficient to offset the effects of chronic toxicity [59]. Exposure to clothianidin dramatically reduced the expression of the AMP genes hymenoptaecin, defensin-1, and abaecin—all of which are critical for defending honeybees against bacterial and fungal infections. The study also showed that exposure to neonicotinoids significantly decreased the expression of dorsal-1A, a crucial transcription factor in the Toll pathway. Because of this suppression, honeybees were less able to combat Deformed Wing Virus (DWV) infections, which resulted in higher viral replication and mortality rates [62]. The exposure to clothianidin and thiamethoxam also interfered with the Toll and Imd (Immune Deficiency) pathways, which are critical for immune signaling and pathogen recognition. The production of antimicrobial peptides was significantly reduced in bees exposed to these pesticides, which hindered their ability to fight off *Paenibacillus larvae*, the organism that causes American foulbrood disease. Bacterial growth within the bee larvae was aided by this suppression, which raised larval mortality and contributed to colony decline [63]. Li et al. (2017) demonstrated that the Prophenoloxidase (PPO) system, which is essential to the melanization immune response, was less active in bees exposed to imidacloprid. Due to this suppression, honeybees were much more susceptible to the midgut infection caused by the microsporidian parasite *Nosema ceranae*, which hinders the absorption of nutrients. In comparison to unexposed controls, the study found that infected bees had higher spore loads and showed higher mortality rates [64].

Exposure to neonicotinoids is also known to cause oxidative stress, which weakens cellular immune responses and further suppresses the immune system. Increased catalase and glutathione-S-transferase activity are signs of oxidative stress caused by honeybee queens’ combined exposure to the neonicotinoid imidacloprid and *Nosema ceranae* infection. Immunosuppression, decreased queen survival, and decreased fecundity are the results of this stress response in conjunction with changed immune gene expression [65]. Brandt et al. (2016) demonstrated that exposure to clothianidin significantly decreased hemocyte proliferation, resulting in a decrease in the number of immune cells available to fight off microbial invaders. This effect was significant even at sublethal doses, indicating that long-term exposure to neonicotinoids, even at environmentally relevant levels, significantly impairs immune resilience [63]. Christen et al. (2016) found that imidacloprid exposure changed the honeybees’ DNA methylation patterns, which resulted in the long-term downregulation of important immune genes. The bees were less able to build immune memory as a result of this epigenetic silencing, which increased their vulnerability to recurring infections [48]. According to the study by Brandt et al. (2016) [63], these alterations may be generational, which could have an impact on honeybees’ future generations and exacerbate population declines. Interactions with other environmental stressors, such as inadequate nutrition, pesticide cocktails, and parasitic infections, exacerbate the immune suppression brought on by neonicotinoid exposure.

**Figure 1:**
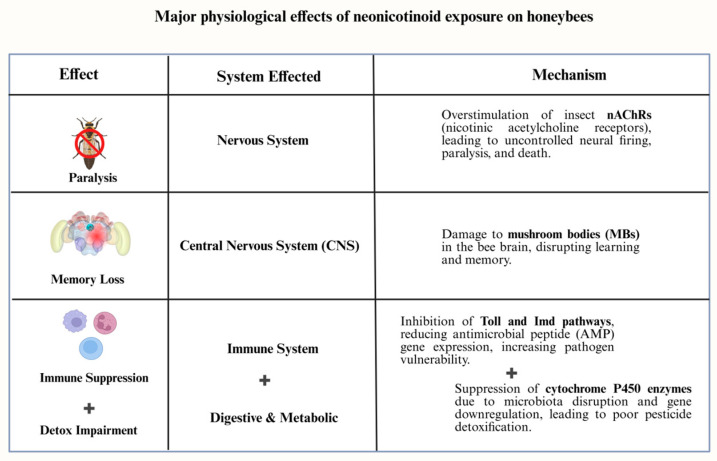
This table summarizes the major physiological effects of neonicotinoid exposure on honeybees across different biological systems.

The relationship between neonicotinoids and *Varroa destructor*, a parasitic mite that impairs honey bee immunity by inhibiting immune gene expression. Bees exposed to neonicotinoids had a markedly increased vulnerability to Varroa mite infestations, which further weaken their defenses against viral infections such as Deformed Wing Virus (DWV) and Israeli Acute Paralysis Virus (IAPV). Varroa infestation and neonicotinoid exposure combined to cause an accelerated colony collapse. Figure 1 summarizes the major physiological effects of neonicotinoid exposure on honeybees across different biological systems.

**Figure 2:**
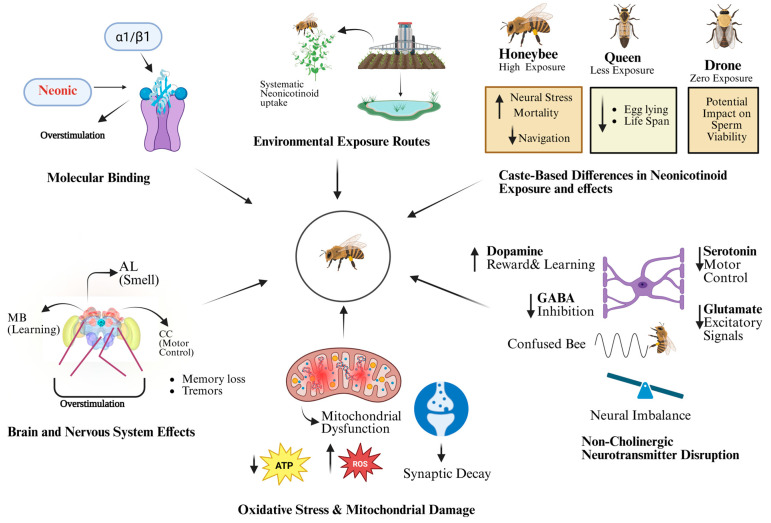
Overview of neonicotinoid effects on honeybee neurobiology. Neonicotinoids bind to nAChRs in the brain, causing overstimulation, neurotransmitter imbalance, oxidative stress, and behavioral impairments like reduced memory, altered waggle dance, and caste-specific effects. Color coding: yellow starburst (ATP) indicates cellular energy production; red starburst (ROS) represents reactive oxygen species and oxidative stress; red shading within mitochondria marks sites of mitochondrial damage/dysfunction; purple neuron graphics depict neurotransmitter signaling pathways; brown/gold boxes highlight caste-specific exposure outcomes (worker, queen, drone).

According to recent research, exposure to compounds like clothianidin and imidacloprid can interfere with key immune signaling pathways such as NF-κB, reducing antiviral defenses and promoting viral replication, especially in honey bees already harboring covert infections, making pathogen impact under certain conditions. Neonicotinoid insecticides have been linked to potential immunosuppressive effects in pollinators, but findings across studies remain inconsistent, warranting cautious interpretation [66]. Bumblebees have also shown similar effects, with high-dose imidacloprid exposure impairing humoral immune responses and phenol oxidase activity, particularly when combined with immunological challenges [67]. Neonicotinoids have also been shown in Drosophila laboratory tests to impair gut immunity by inhibiting Duox-mediated hydrogen peroxide synthesis, which leads to dysbiosis and reduced resistance to infections [68]. However, despite significant changes in gene expression, some studies have found minimal effects on physiological immune responses, suggesting that the relationship between transcriptional alterations and true immunological competence is not clear-cut [69]. This diversity is also evident in field trials; for example, extended exposure to neonicotinoids under actual agricultural conditions decreased colony survival and social immunity when paired with fungicides, but other scenarios produced less noticeable results [3]. Although further research is needed to confirm this theory, mechanistic evidence suggests that acetylcholine-based signaling in hemocytes may be disrupted with, indicating a new route for immunological regulation [70]. So, species differences, and the role of additional stressors, even though there is solid evidence that neonicotinoids may reduce immunity under specific environmental conditions, doses, and co-stress scenarios. Figure 2 illustrates how neonicotinoids affect the honeybee brain, from receptor binding to behavioral changes.

## 3. Effect of Neonicotinoids on the Development of Honey Bees

**Figure 3:**
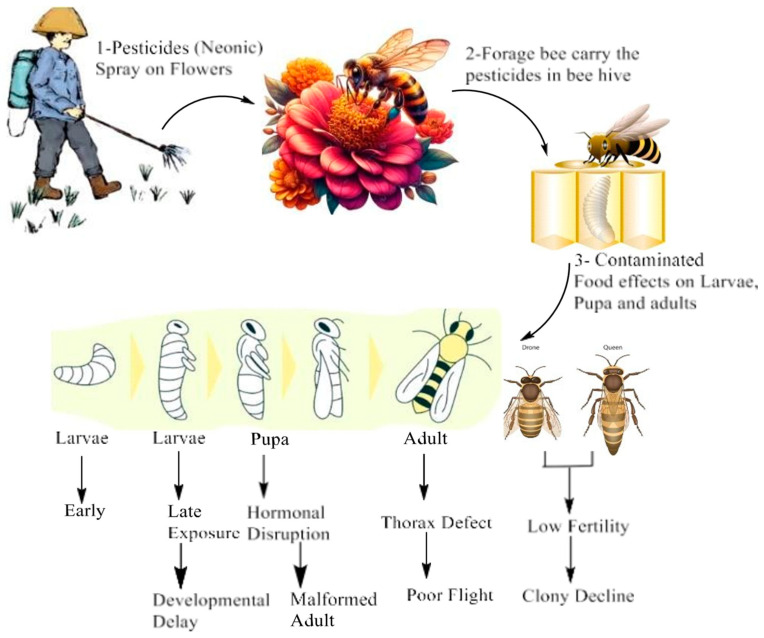
Impact of neonicotinoid pesticides on honeybee colonies. Pesticides sprayed on flowers are collected by foragers and brought back to the hive, contaminating food shared with larvae, pupae, and adults. This leads to developmental delays, malformations, flight defects, reduced fertility, and ultimately colony decline.

One of the most important issues with honeybee health is the bioaccumulation of neonicotinoids in royal jelly and brood food, which directly affects pesticide exposure in larvae. Pesticide residues are transferred to the brood and consumed directly by foraging bees. Morfin et al. (2019) showed that when bees consumed neonicotinoid-treated plants, the chemicals’ concentrations in the food sources increased and were transferred to the larvae, resulting in a substantial exposure pathway. Additionally, neonicotinoid contamination of royal jelly had a major impact on larval development [71]. Ben Abdelkader et al. (2021) reported that royal jelly, a vital food source for growing larvae, contains both pesticide residues and nutritional components that may lessen or increase the toxicity, which further complicates the bioaccumulation process. These studies demonstrate how pesticides build up within the hive and travel up the food chain to reach larvae at crucial developmental stages [72]. Fisher and Rangel (2018) further showed that the neurological disruption brought on by the pesticides is linked to this delayed development because it hinders the larvae’s capacity to properly process nutrients and grow [73]. Similarly, Tosi and Nieh (2019) [13] discovered that exposure to thiamethoxam led to higher larval mortality rates, with impacted bees failing to reach the pupal stage. Additionally, larvae exposed to sublethal pesticide concentrations displayed signs of decreased feeding responses, which ultimately resulted in starvation. The neurological damage brought on by the pesticide was the cause of the poor feeding behavior, demonstrating that exposure to pesticides impairs fundamental physiological processes such as feeding, which are essential for survival and growth. Ben Abdelkader et al. (2021) noted that mortality rates varied depending on when exposure occurred, during early or late larval stages. While later-stage exposure caused developmental delays, early-stage exposure increased mortality. This variation implies that the timing of the pesticide exposure is critical, with early exposure leading to irreversible developmental problems [72]. Additionally, Pereira et al. (2020) reviewed multiple studies that demonstrating the sublethal impacts of thiamethoxam on the pupal metamorphosis and development of *Apis mellifera*. In one study, A. mellifera larvae were exposed to 0.001 ng/μL of thiamethoxam, which resulted in no significant difference in pupal survival. Another study exposed larvae to a sublethal concentration of 1.43 ng/μL, which caused condensed cells and early cell death in the optic lobes of pupal brains, indicating disrupted neurodevelopment and early degeneration of brain tissue. Furthermore, they evaluated stress biomarker activity following exposure to three doses of thiamethoxam: 0.00001 ng/μL, 0.001 ng/μL, and 1.44 ng/μL, and found increased activity of acetylcholinesterase (AChE), carboxylesterases (CaE), and glutathione S-transferase (GST) specifically in the pupal stage, indicating that oxidative stress was triggered and potentially harmed pupal physiology [74]. The same study by Tavares et al. (2019) also noted that synapsin protein levels, which are critical for synaptic function in the nervous system, were reduced in both pupae and newly emerged adult bees after larval exposure to thiamethoxam [75]. Figure 3 is a graphical summary of this extensive number of developmental disruptions that neonicotinoids cause at several points throughout the lifecycle of a honeybee: Fed on contaminated food, larvae do not develop normally; pupae are unable to emerge; and adults that emerge fail to reproduce, eventually causing their colony to decline.

### Reproductive Toxicity in Queens and Drones

Sublethal exposure to neonicotinoid insecticides, specifically thiamethoxam and clothianidin, significantly reduces sperm viability and quantity in male honey bees (*Apis mellifera*). The study found that drones exposed to field-realistic doses of these neonicotinoids exhibited approximately a 39% reduction in the number of living sperm compared to unexposed controls. Neonicotinoid exposure significantly reduces sperm viability in honey bee drones. Additionally, the pesticides impair drone flight and mating behaviors, which likely lowers their reproductive success [76]. The exposure to field-realistic concentrations of neonicotinoid pesticides during queen development significantly impairs the reproductive health and longevity of honeybee queens. The study found a 20% reduction in stored spermatozoa and a 9% decrease in sperm viability in queens exposed to neonicotinoids, compared to controls. Moreover, a 34% decline was observed in the number of queens that survived and produced worker offspring after four weeks [77]. Queens developed in colonies exposed to pesticide-treated pollen produced fewer viable queens compared to controls. Among the surviving queens, sperm viability was reduced, and the colonies they established exhibited lower brood viability. Despite royal jelly containing minimal pesticide residues, these negative effects on queen reproductive quality are likely indirect, possibly resulting from altered social interactions within the colony [78]. Chaimanee et al. (2016) [79] studied the effects of exposure to the neonicotinoid insecticide imidacloprid and the organophosphate acaricide coumaphos on honey bee queens (*Apis mellifera*). Their results showed that exposure to these chemicals significantly reduced sperm viability in queen spermathecae, which is critical for sustaining long-term fertility. The reduction in sperm viability was linked to altered gene expression related to immune and stress responses in queens.

## 4. Effects on Adult Longevity

### 4.1. Neonicotinoid-Induced Cellular Damage and Aging

Neonicotinoids like imidacloprid, thiacloprid, and clothianidin impose harmful impacts on honey bees (*Apis mellifera*) through interfering with nervous communication pathways as well as cellular metabolism. These pesticides function as activators of nicotinic acetylcholine receptors (nAChRs) inside the bee brain, resulting in prolonged nervous stimulation and eventually nerve damage [80]. Excessive stimulation of nAChRs has been proven to interfere with control of crucial neurotransmitters, decreasing neural plasticity along with weakening learning and memory abilities. Such continuous overstimulation of neural pathways speeds up age-associated mental impairment, making it more difficult for bees to interpret environmental signals, and remember the acquired actions [81]. Along with neurological harm, extended exposure to neonicotinoids has been associated with DNA damage and defective cellular recovery mechanisms. At the molecular level, neonicotinoids interfere with genetic regulation pathways that are responsible for toxic removal and oxidative stress response, resulting in elevated susceptibility to ecological contaminants. The accumulated harm resulting from oxidative stress weakens the neurotransmission and programmed cell death that leads to reduced longevity and impaired immune protection in honeybees [51]. Recent research from shows that exposure to pesticides, especially neonicotinoids like imidacloprid, causes *Apis mellifera* bees experience severe oxidative stress, which compromises mitochondrial and antioxidant defenses. Imidacloprid exposure in the field resulted in increased levels of nitrite and protein carbonyl, decreased activity of catalase (CAT) and superoxide dismutase (SOD) in larvae, and structural damage to the midgut and fat body, endangering the health of the colony [82]. The expression of important mitochondrial (MnSOD) and cytosolic (CuZnSOD) antioxidant enzymes, as well as the enzyme that produces hydrogen peroxide (DUOX), was also affected by combined exposure to imidacloprid and amitraz, indicating that pesticides interfere with immune-linked ROS pathways [83]. There are not many studies on melatonin supplementation in bees under pesticide stress, but information from other biological models shows that it has a lot of potential as a countermeasure. In mung bean seedlings under cadmium stress, melatonin improved oxidative defense and energy status by increasing SOD, CAT, and other TCA cycle enzymes and improving mitochondrial energy metabolism, increasing ATP synthase activity by 63.6% [84]. Similarly, 100 µM melatonin enhanced photosynthetic efficiency, osmolyte accumulation, and antioxidant enzyme activity in heat-stressed buckwheat [85]. Melatonin, on the other hand, reduced hydrogen peroxide levels and increased the expression of the SOD and CAT genes in maize and wheat, thereby mitigating the effects of salinity and drought stress [86,87]. These results support melatonin’s use as a biological agent to improve pollinator resilience by showing that its antioxidant and mitochondrial-protective qualities directly address the oxidative and energetic deficits observed in bees exposed to pesticides.

Neonicotinoids have been proven to trigger mitochondrial impairment via suppressing oxidative phosphorylation and lowering ATP synthesis, which is essential for cellular energy production [80]. Mitochondria are necessary for energy homeostasis in the bees, especially for maintaining extended flight behavior and thermoregulation inside the beehive. Nevertheless, exposure to sublethal levels of thiacloprids has been associated with substantial rises in reactive oxygen species (ROS), resulting in lipid oxidation and protein breakdown in honeybee units [88]. Additional research shows that oxidative stress indicators like superoxide dismutase (SOD) and catalase (CAT) are considerably increased in neonicotinoid-treated bees, indicating that these insecticides trigger a condition of persistent physiological stress. Consequently, bees suffer from decreased cellular strength and an impaired ability to recover metabolically, resulting in early aging and elevated mortality levels [81]. Lipid metabolism serves a crucial role in energy storage and consumption in honeybees especially for foraging, flight resistance, and colony management. Yet, subjection to imidacloprid and thiacloprid has been proven to disturb lipid homeostasis, leading to decreased fat body stores and elevated energy depletion. Bees suffering from lipid metabolism imbalance display a lower energy store, making them more vulnerable to temperature-related stress, starvation, and reduced fighting efficiency [88]. Furthermore, research conducted by Tsvetkov et al. (2017) [3] discovered that imidacloprid-contaminated pollen caused a 20% decline in glucose metabolism, even more intensifying energy deficit in worker bees. These observations indicate that neonicotinoids lead to an extensive metabolic disruption, eventually compromising honeybee colony stability.

### 4.2. Impact on Worker Bee Survival Rates

Even at reduced environmental levels, prolonged interaction with neonicotinoids has been observed to notably decrease the longevity of worker bees [89]. Field research indicates that the colony subjected to neonicotinoid-treated crops faces increased mortality rates, along with worker bees displaying a reduction in lifespan in contrast to untreated hives [3]. The major cause leading to this decrease is impaired immune function, as neonicotinoid-treated bees display a greater vulnerability to bacterial and fungal infections, especially *Nosema ceranae*. Moreover, experimental tests have shown that neonicotinoid-treated bees display a modified cleaning behavior contributing to decreased pathogen resistance and heightened entire colony transmission of disease. Such observations indicate that not only instantly decrease worker bees’ survival duration, But also, as a secondary effect, it speeds up colony collapse by impairing the entire well-being of the bee population [90]. Neonicotinoids react collectively alongside various environmental stressors, intensifying their harmful impacts on honey bee health. Inadequate nutrition increases honeybee susceptibility to neonicotinoid toxicity by compromising their detoxification systems and diminishing their ability to metabolize harmful compounds efficiently [91]. Moreover, temperature variation enhances the neonicotinoid toxicity, with cold-exposed bees facing reduced pesticide metabolism, bringing about extended harmful impacts. Experiments have indicated that environmental stressors such as habitat destruction, along with environmental fluctuations, additionally intensify the adverse impacts of neonicotinoids, causing increased overwintering death rates [81]. Moreover, Doublet et al. (2015) observed that bees subjected to both neonicotinoids and *Nosema ceranae* infections display a death rate twice as great as those exposed to solely a single stressor, illustrating that pesticide reduces immune defenses, making bees further susceptible to pathogens [90].

## 5. Behavioral Effects of Neonicotinoids on Honey Bees

### 5.1. Neural Basis and Impairment of Learning and Memory

Different neural structures tightly regulate the behavior of honey bees, including learning, memory, and navigation. The mushroom bodies play a key role in memory formation and sensory integration, exhibiting high neuroplasticity associated with behavioral changes such as foraging [92]. As the main olfactory processing organs, the antennal lobes are essential for social interaction and odor perception [34]. The central complex integrates multisensory cues to direct movement and is important in spatial orientation and navigation [93]. Key neurotransmission is mediated by nicotinic acetylcholine receptors (nAChRs), but neonicotinoids overstimulate these receptors, impairing olfactory learning, failing synaptic pruning, and causing long-term memory problems [94]. By focusing on particular brain circuits essential to memory, navigation, and circadian regulation, neonicotinoids disrupt honey bee behavior. These substances overstimulate nicotinic acetylcholine receptors (nAChRs) in the mushroom bodies, which are important sites for memory formation. This causes an excessive input of calcium, which interferes with signaling cascades that are necessary for long-term memory. In memory circuits, this interference lowers the activity of transcription factors linked to memory, such as CREB and CaMKII, which causes synaptic malfunction and even cell death [95]. Simultaneously, decreased cellular energy metabolism and disturbed neuronal signaling both contribute to flight disorientation. When exposed to neonicotinoid, mitochondrial genes including cox5a and cox17 are downregulated, which impairs ATP synthesis and reduces the amount of energy available for prolonged flight. The longer and less effective homing flights seen in exposed bees are directly related to this metabolic stress [96]. Additionally, these pesticides cause phase shifts in behavioral rhythms, disturbed sleep, and decreased synchronization of activity patterns with ambient light cycles by abnormally stimulating cholinergic inputs to clock neurons. These disturbances affect navigation and time-memory, two processes necessary for effective foraging and colony coordination [97].

The proboscis extension response (PER) is an extensively applied test to evaluate associative learning in honey bees. In normal conditions, bees link a particular scent with a sucrose reward learning and their proboscis with the odor. On the other hand, subjection to the neonicotinoid pesticide considerably disrupts their learning ability. Williamson and Wright (2013) have shown that bees exposed to several cholinergic pesticides, such as imidacloprid and clothianidin, demonstrated a decreased PER performance. These bees demanded notably more trials to form a connection, and many were unable to preserve the learned response [98]. Likewise, Mustard et al. (2020) found that honey bees subjected to neonicotinoid-infused nectar were unable to differentiate among floral odors in learning tasks, resulting in a 40% reduced success rate regarding foraging perception [99]. Such perceptions suggested by Siviter et al., 2018 are that neonicotinoids modify neural pathways connected with associative learning as well as neural processing, making it more challenging for bees to search for food and identify earlier advantageous floral sources [81]. Piiroinen and Goulson (2016) examined how the long-term exposure of neonicotinoids affects cognitive processing and learning speed in honeybees and bumblebees. Their results validated that subjected bees needed notably more practice trials to link a reward with an adapted stimulus. Such postponement in learning decreases foraging performance and can result in weaker colony stability [100]. Additionally, Siviter et al. (2018) measured the degree of cognition impairment because of neonicotinoids and also validated that honeybees demanded 50% more training sessions to acquire basic tasks, in contrast with control bees [81]. Another study by Wright et al. (2015) observed that despite the low doses of neonicotinoids, impaired short-term memory caused bees to neglect the earlier learned floral choices. This caused affected honeybees to roam among various flower types instead of focusing on high-reward flowers, eventually resulting in decreased pollen and nectar gathering [101]. Recent studies show that imidacloprid alters brain gene expression, affecting energy metabolism, neurotransmitter systems, and memory pathways, leading to impaired memory and spatial orientation in honey bees [102]. Paoli and Giurfa (2024) revealed that neonicotinoids disrupt the neural functions in mushroom bodies that regulate cognitive processing. Through interfering with their vital cognitive pathways, bees experience challenges in maintaining acquired skills, thereby decreasing their efficiency in finding the floral resources and sharing the essential signals with their nestmates [34]. Favaro et al. (2022) discovered that bees constantly subjected to imidacloprid and clothianidin displayed reduced sensory reactions in response to typical flower compounds. This dysfunction in signal processing ability creates a challenge for bees in selecting a rich pollen supply, resulting in ineffective food gathering [103]. Mustard et al. (2020) additionally verified that bees were unable to distinguish among the floral odors after ingesting a neonicotinoid-induced nectar, resulting in disorganized foraging patterns and wasted energy on unprofitable blooms. Gradually, this repeated ineffectiveness decreases the colony-level resource consumption, leaving the colony more vulnerable to starvation as well as destruction [99]. Ke et al. (2023) highlighted that thiacloprid exposure impaired cognitive processes in these brain regions, resulting in weakened olfactory sensory interpretation. Consequently, bees could not establish long-term floral-based odor recognition, lowering their capacity to relocate nutrient-rich food sources effectively [104]. Furthermore, Belzunces et al. (2012) observed that neonicotinoid exposure disrupted the brain-related reactions in the antennal lobe, interfering with the process by which bees interpret and react to pheromonal and floral stimuli. This alternation can result in a misunderstanding of flower indicators, foraging mistakes, and reducing the entire colony’s success [105]. Kessler et al. (2015) observed that neonicotinoid-exposed bees exhibited impaired flower-related consistency, often alternating among various flower varieties instead of preferring a specific rewarding variety. This action decreases the pollination efficiency and enhances hive energy consumption [106].

### 5.2. Navigation, Foraging Behavior, and Flight Dysfunction

Fischer et al. (2014) observe that exposure to neonicotinoids displays a weakened flight accuracy with an increased chance of being unable to navigate back to their nests post-foraging. This was associated with disturbance in the mushroom body neural pathway, which is crucial for interpreting spatial memories and orientation signals [107]. Likewise, Tison et al. (2016) stated that prolonged exposure to thiacloprid caused a persistent dysfunction in homing ability. Bees displayed a prolonged navigation duration and reduced orientation accuracy, indicating that the pesticides disturb the neural circuits essential for spatial cognition [88]. In another study Stanley et al. (2016) analyzed the effect of field-realistic interaction with neonicotinoids on bumblebees’ foraging, and bees experienced trouble in finding their colony after foraging journeys, revealing neurotoxic disruption with cognitive retention [108]. Tosi et al. (2017) proved that honey bee exposure to thiamethoxam displayed a decreased wingbeat frequency and limited flight duration compared to untreated bees. The research determined that flight capability is substantially reduced when bees consume even a low dose of pesticides [109]. Moreover, Williamson and Wright (2013) discovered that subjection to neonicotinoids altered the motor function of adult worker honeybees, decreasing their capacity to maintain long-distance aerial foraging trips. Muscular dysfunction resulted in decreased pollen and nectar accumulation that eventually reduced the colony’s strength. Affected bees also showed erratic flight routes and uncoordinated movements reflective of neuromuscular disorder triggered by pesticide interference with acetylcholine receptors [98].

### 5.3. Disruption of Circadian Rhythms and Social Communication

Tackenberg et al. (2020) identified that honey bees subjected to neonicotinoids displayed impaired circadian gene expression, leading to a modified sleep-wake cycle and foraging timing. This impairment decreases hive productivity and communication among worker bees, resulting in inefficient pollen and nectar collection methods. Circadian dysfunction not only alters the timing of foraging trips but also affects learning and memory maintenance, making it more difficult for bees to identify ideal foraging periods and floral resources [110]. Bloch et al. (2017) examined the correlation between the circadian clock in bees and the flowers, showing that neonicotinoid exposure lowered the time perception and postponed the identification of the most effective foraging periods. Bees subjected to pesticides exhibited a slowed morning foraging pattern and erratic pollen collection patterns that adversely impacted the plant-pollinator coordination [111]. Traynor and Lamas (2021) discovered that beehives subjected to sublethal levels of neonicotinoid-laced pollen encountered disrupted trophallactic exchanges, leading to disturbance in larval feeding and lowered brood development. The research also found that the worker bees displayed infrequent feeding exchanges, indicating that neonicotinoid disrupts the social cohesion and collaboration in the colony [112]. Another study by Eiri and Nieh (2012) revealed that honeybees exposed to neonicotinoids displayed a shortened dance time, erratic movement patterns, and reduced recruitment effectiveness. This disruption leads to fewer worker bees being effectively guided to resource areas, resulting in food shortages in the hive [113]. Li et al. (2020) discovered that this resulted in honeybees overstimulating or inaccurately estimating distances when signaling foraging locations, decreasing the efficiency of recruiting foragers [114]. Eiri and Nieh (2012) observed that worker bees exposed to neonicotinoids did fewer waggle dance circuits, contributing to lowered recruitment efficiency and decreased nectar gathering. This research highlights how pesticides that induce disturbances in spatial awareness can notably impair colony efficiency [113]. Additionally, Crall and Raine (2023) showed that neonicotinoid treatment impacts coordination among the worker bees along with colony workers, resulting in slower recruitment response and reduced collective foraging efficiency. This failure of coordination in the colony leads to an inability to effectively utilize food resources and eventually reduces the colony’s productivity and heightens the risk of death [115]. Recent research unequivocally shows that memory, navigation, and foraging behavior deficits brought on by neonicotinoid use might lower pollination effectiveness and jeopardize colony survival. For instance, thiacloprid treatment dramatically impairs honey bees’ memory and learning by changing gene expression and causing neuronal apoptosis, which in turn impairs their capacity to remember flower cues and return to the hive [116]. 

Similarly, bees’ ability to recognize flowers, which is crucial for effective foraging and recruitment, is diminished by thiamethoxam-induced deficiencies in odor discrimination and complex learning [99]. It has been demonstrated that sublethal imidacloprid treatment significantly lowers pollen foraging at the colony level, which has an impact on colony growth and brood nutrition [117]. Furthermore, neonicotinoid exposure causes circadian rhythm abnormalities that worsen social communication and time-memory, which are crucial for coordinated foraging [110]. When combined, these neurobehavioral disturbances impair brood care, decrease foraging efficiency, and jeopardize honey bee colonies’ long-term viability.

## 6. Colony-Level Consequences and Ecological Implications

### Reduced Colony Growth and Population Decline

By affecting foraging behavior, lowering worker longevity, and changing brood rearing patterns, neonicotinoids—in particular, imidacloprid, thiamethoxam, and clothianidin—are known to interfere with colony development. Colony size and general vitality significantly decrease after prolonged exposure to neonicotinoids. Imidacloprid concentrations as low as 5–10 ppb have been shown in experiments to cause decreased food intake and foraging activity, which in turn slows colony growth and reduces brood development [80]. Colonies given field-realistic dosages of thiamethoxam showed markedly reduced brood survival and developmental delay in field settings, which impeded population replenishment [118]. Additional research demonstrates that extended exposure to sublethal neonicotinoid concentrations lowers larval survival, which affects colony sustainability over several generations [119]. Seasonal neonicotinoid exposure reduces worker bee lifespan, which destabilizes the colony’s social structure and increases the chance of collapse, according to a predictive mathematical model of honeybee colony dynamics [120]. According to Blacquiere et al. (2012), worker bees exposed to sublethal doses of imidacloprid exhibit delayed foraging behavior and spend less time gathering pollen, which is essential for the nutrition and development of brood [80]. Imidacloprid sublethal exposure at 1.5 ppb reduced successful foraging trips by 23% in a field trial because of memory dysfunction and impaired navigation [118]. Similarly, studies using sucrose solutions treated with thiamethoxam showed that exposed bees were less able to identify familiar floral scents, which reduced their efficiency in acquiring food [119]. Furthermore, by inhibiting the waggle dance, a crucial behavior for alerting coworkers to food locations, neonicotinoids undermine social communication within the colony, which further reduces foraging success and resource collection [120]. Field tests by Alburaki et al. (2015) revealed that colonies of honey bees (Apis mellifera) situated close to cornfields treated with neonicotinoid showed noticeably more pathogen infections than colonies near untreated fields. Bees foraging on treated crops showed increased acetylcholinesterase (AChE) activity, and the study found a positive relationship between neonicotinoid exposure and pathogen load [121]. Godfray et al. (2015) pointed out that a number of interrelated stressors, such as pathogens, parasites, and pesticide exposure, contribute to declines in Apis mellifera [91]. Blacquiere et al. (2012) noted that there was no evidence of a direct causal relationship between neonicotinoids and increased susceptibility to pathogens. To promote the best possible development of their brood, honeybee colonies strictly regulate the temperature inside their hives. Exposure to neonicotinoids disrupts this function, leading to erratic hive temperatures and elevated brood mortality. Imidacloprid exposure has been shown to reduce worker bee thermoregulatory responses, resulting in less-than-ideal temperatures for larval development [80]. Neonicotinoid-treated colonies in a 16-week chronic exposure study were unable to maintain thermal stability in colder temperatures, which resulted in delayed brood development and a lower pupation success rate [118]. Clothianidin at sublethal doses changes nurse bee behavior, decreasing the frequency and efficiency of larval feeding and raising the rate of brood abandonment, according to laboratory tests [119]. Furthermore, thiamethoxam-exposed colonies demonstrated a notable decrease in brood comb construction, with more than 80% of treated hives generating insufficient combs to sustain upcoming worker generations [120]. For the colony to reproduce and survive over the long term, the queen bee’s productivity and health are essential. By decreasing egg-laying rates and increasing queen replacement events, neonicotinoids disrupt queen function. The dynamics of colony populations were directly impacted by experimental evidence showing that queens exposed to imidacloprid at 10 ppb for an extended period of time laid 30% fewer eggs than their counterparts who were not exposed [80]. In colonies exposed to neonicotinoids, frequent queen supersedure was noted, which disrupted brood production and made it more difficult for the colony to sustain a healthy workforce [118]. It has also been demonstrated that neonicotinoids reduce drone fertility; treated drones show reduced sperm motility and viability, which lowers the likelihood of mating and subsequent queen fertilization [119]. Drone bees reared in clothianidin-exposed colonies demonstrated significant declines in reproductive capacity, which poses long-term risks to colony genetic fitness and sustainability [120].

Neonicotinoid exposure may have the most disastrous effect because it may be linked to Colony Collapse Disorder (CCD), a condition in which worker bees abruptly leave a hive, leaving behind the queen, brood, and food supplies. High levels of neonicotinoid usage are strongly associated with an increased incidence of CCD in different geographic regions, according to a synthesis of global monitoring reports from 2006 to 2015 [80].

Pesticides continue to play an associative rather than a definitively causative role in colony collapse disorder (CCD), which is now generally acknowledged as a multifactorial syndrome rather than the result of a single cause. Although these effects are highly context-dependent and frequently seen at sublethal doses in laboratory settings rather than in real-world settings, recent research highlights that pesticides, especially neonicotinoids, may exacerbate CCD by compromising immune responses and raising susceptibility to pathogens [122]. Furthermore, synergistic stressors such as Varroa destructor infestations, fungal and viral infections, dietary deficiencies, and extreme weather conditions are closely associated with CCD events [123]. There is proof that exposure to pesticides frequently makes viruses like the Deformed Wing Virus (DWV) more likely to replicate when the immune system is suppressed [124]. Crucially, the majority of results are still correlational because extensive field research frequently cannot reproduce the dramatic colony losses observed in controlled environments, indicating that CCD is rarely brought on by pesticide exposure alone [125]. Further confounding causal attribution, new models and epidemiological surveys show that habitat stress and extreme weather events are equally important in colony loss patterns [126]. There is a need for integrated, long-term research that look at pesticide impacts in combination with diseases, nutrition, and environmental factors because the evidence often points to interacting pressures rather than a single component as the cause of CCD.

Repeated seasonal exposure to imidacloprid causes cumulative worker bee losses, which ultimately push colonies below critical thresholds for survival and cause irreversible collapse, according to long-term field studies [118]. Colony failure rates are higher among beekeepers working in heavily farmed landscapes, indicating that exposure to pesticides at the landscape level is a major factor contributing to continuous population declines [120].

A number of long and extensive studies carried out indicate there is little or no adverse effect on the survival and productivity of honeybee colonies against exposure to neonicotinoids at field-realistic levels. As example, Flores et al. (2021) observed that adult bees and brood development did not differ significantly between colonies exposed to thiamethoxam and clothianidin and controls, with differences only persisting during exposure [127]. The same could be seen in Meikle et al. (2021, 2022), who found that colony metabolic activity (e.g., increased brood production and higher CO_2_ levels) was stimulated by low levels of imidacloprid but did not increase productivity, or impact survivorship [128]. Additional studies show that the effect of neonicotinoids alone on colonies is not significant unless actuated by other stresses such as Varroa mite infestations [129]. The result of all these studies is an indication that based on some environmental and experimental conditions, neonicotinoids might affect the colony-level metrics of the honeybee in neutral or even a little stimulatory way.

## 7. Mitigation Strategies and Future Perspective

### 7.1. Reducing Honeybee Exposure to Neonicotinoids

A study by Tosi et al. (2017) founded that honeybees that were exposed to neonicotinoids and nutritional stress simultaneously experienced a synergistic decline in survival rates. During crucial times, like the flowering season, when foraging bees are most likely to come into contact with contaminated nectar and pollen, honeybees’ exposure to these chemicals must be minimized. The authors suggest timing restrictions for pesticide application during this time to limit exposure to dangerous chemicals based on their findings. So, honeybee exposure to neonicotinoids can be reduced by using integrated pest management (IPM) techniques that include less harmful pest control techniques like crop rotation and biological pest control. Research on the application of biopesticides as a practical substitute for synthetic neonicotinoids is crucial [130]. Several biological alternatives, such as bacterial formulations, fungal-based biopesticides, and plant-derived insecticides, were emphasized by Smagghe et al. (2023) as alternatives that are less hazardous to non-target species like honeybees. Their research supports the use of biological control agents, cultural practices, and biopesticides in integrated pest management systems. In addition to decreasing reliance on dangerous neonicotinoids, these tactics shield honeybee populations from exposure to chemical residues. They also did point out that although biopesticides show promise, more investigation is necessary to ascertain their long-term effectiveness in extensive agricultural systems. For IPM systems to be developed effectively, biopesticides must be compatible with conventional insecticides [131].

Although it is accepted that biopesticides are safer, and environmentally sustainable, there are high technical, economic, and institutional barriers that hinder large-scale use of biopesticides. The issues of cost and economic feasibility have remained the main challenges; biopesticides are usually more costly to produce and formulate compared to synthetic ones and as such, they are not beneficial to resource-seeking farmers [132]. In addition to this, the unstable shelf-life inhibits penetration into the market, as products of microbial origin are easily degraded by high temperatures and moisture (typical of tropical storage environments), resulting in inconsistent effectiveness [133]. Most products have poor performance and delayed pest mortality in the field even after being stabilized which fails to inspire farmers to trust them [134]. Current regulations that target synthetic pesticides lead to the provision of insurmountable approval methods, which add to the ballooned cost of development by further delaying commercialization [135]. Because of knowledge gaps among farmers and the absence of an organized extension service, this adds to the issue leading to little awareness and inappropriate use, which reduces the perception of efficacy [136]. Although innovations involving technologies such as microencapsulation and UV-protective coating on pharmaceuticals appear to have potential to enhance persistence and decrease frequency of application [137], there are crucial gaps in scale and costs. These concerns represent critical areas in which policymakers need to focus on the issue of biopesticide regulation and the reform of policy, enhanced formulations, and training improvements to facilitate the transition of biopesticides from niche application to a mainstream element of integrated pest management [138].

Kopparthi (2020) asserts that microbial-based formulations and biopesticides such as Bacillus thuringiensis can be combined with traditional insecticides without having a major negative effect on honeybee populations. This preserves the effectiveness of pest control while enabling farmers to rely less on harmful neonicotinoids. To evaluate the safety and compatibility of biopesticides when used in conjunction with other chemicals, they also underlined the significance of field trials. His research recommended the creation of biopesticide blends that minimize negative impacts on non-target species, such as honeybees, while targeting a wider variety of pests. Another crucial element in reducing the detrimental effects on honeybees is the regulation of neonicotinoid use [139]. In their review of neonicotinoids’ environmental hazards, Hladik et al. (2018) emphasized the necessity of stricter regulations to control exposure. According to their research, nations such as the European Union have already taken action to limit the use of neonicotinoids, especially in regions that are essential for pollinator habitats. According to that, prohibitions on neonicotinoid use in flowering crops, where honeybees are most likely to come into contact with pesticides, should be part of any effective regulation. To guarantee that agricultural productivity is not jeopardized, they emphasized that research into alternate pest management techniques must be conducted in addition to these regulatory measures [140]. Demortain (2021) critically analyzed how ecotoxicological studies influenced EU neonicotinoid regulations. The paper emphasizes how, even before complete agreement had been reached, scientific evidence of sublethal effects, like behavioral disruption and impaired navigation in honeybees, catalyzed policy changes. This represents a significant departure from conventional regulatory models in favor of a preventative framework that proactively addresses new risks. They also emphasize how interdisciplinary coalitions played a crucial role in converting lab results into regulatory discussions, which ultimately resulted in the European Union banning some neonicotinoids [141]. 

The nutrition of honeybees is one of the less discussed facets of their defense. The importance of varied forage resources in preserving honeybee health was highlighted by Tosi et al. (2017) who discovered that honeybees fed a nutritious diet were more resistant to the harmful effects of neonicotinoids. According to the study, pollinator-friendly farming methods, like growing flowering plants and crops high in nectar, may strengthen honeybee immunity and improve their capacity to detoxify chemicals [130]. Smagghe et al. (2023) claimed that the development of sustainable pest control alternatives depends on ongoing research into low-toxicity insecticides. Additionally, they pointed out that bioengineered pest-resistant crops may reduce the need for chemical treatments, which would reduce the chance that honeybees would be exposed to pesticides [131].

Furthermore, to fully comprehend the effects of alternative pest control techniques on honeybees and other pollinators, Jactel et al. (2019) underlined the significance of long-term ecological monitoring. Their study emphasized the need for field tests in the future to assess the ecological effects and efficacy of biopesticides and alternative chemical formulations in practical environments [142].

### 7.2. Integrated Pest Management (IPM) and Sustainable Agriculture

In a systematic review, Lundin et al. (2015) examined the function of IPM in reducing the need for neonicotinoids in their systematic review. According to the study, honeybee exposure to hazardous chemicals could be considerably decreased by using integrated pest management (IPM) strategies that combine cultural practices, biological control techniques, and low-toxicity insecticides. IPM systems provide long-term pest control solutions that are good for pollinators in addition to being a sustainable substitute for methods that heavily rely on pesticides. Additionally, the authors stressed the significance of promoting the adoption of sustainable pest management techniques that put pollinator health first and educating farmers about the advantages of integrated pest management [143]. Neonicotinoid regulation is persuasively criticized by Kurwadkar and Evans (2016), who contend that the systemic nature of these chemicals has resulted in extensive and ongoing environmental contamination. Pollinators are exposed to these pesticides through a variety of indirect pathways because they penetrate plant tissues and persist in soils and water bodies for a long time after application. The authors call the regulatory reliance on acute toxicity thresholds a systematic failure that fails to consider synergistic and chronic risks. Their study urges a revision of existing risk assessment frameworks and fervently supports integrated pest management (IPM) as a sustainable substitute [144].

### 7.3. Emerging Mitigation Strategies

**Figure 4:**
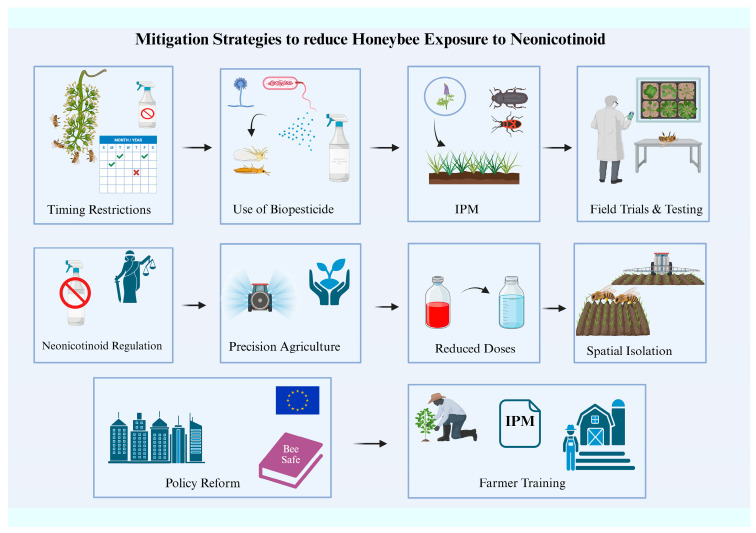
Strategies to mitigate honeybee exposure to neonicotinoids include integrated pest management (IPM), utilizing low-toxicity biopesticides, implementing restricted use during flowering, and enhancing bee nutrition and forage diversity. Arrow coding: Black arrows indicate the sequential flow of steps; turned arrows highlight specific targeted action on the insects.

A number of new tactics are being acknowledged for their efficacy in lowering honeybee exposure to neonicotinoids, in addition to biopesticide substitution and integrated pest management (IPM). Habitat restoration and enhancement, which includes the establishment and maintenance of pollinator-friendly habitats such buffer zones, hedgerows, and wildflower strips, is one of the most promising strategies. Particularly in regions where farmland predominates, these natural landscapes serve as barriers to chemical drift in addition to providing uncontaminated fodder, hence lowering exposure [145]. Furthermore, solutions for reducing off-target pesticide application have evolved in the form of precision agriculture technologies, including as AI-based monitoring systems and GPS-guided spraying. Even sublethal concentrations of neonicotinoids dramatically limit pollen foraging activity, according to studies utilizing AI-assisted models, highlighting the significance of focused, regulated chemical application [146]. Furthermore, since scientists discover that pollinators are regularly exposed to a broad range of agrochemicals, not just neonicotinoids, chemical substitution and lower pesticide doses are becoming more popular. The cumulative dangers to bees can be significantly decreased by using less toxic substances or using smaller dosages [147]. Limiting direct interaction with pesticides can also be achieved through temporal and spatial isolation, such as changing planting schedules or physically separating treated and pollinator-attractive crops. Monitoring studies, for instance, showed geographical heterogeneity in pesticide contamination in pollen and nectar, bolstering planting site and time management techniques [148]. Additionally promising are developments in bee-protective pesticide formulations, including polymer coatings and stereoisomeric chemicals. Honeybee metabolic and detoxification pathways have been discovered to be less disrupted by modified chemical structures, indicating a new approach for safer pesticide production [149]. In addition, bees may be protected from pesticide-induced stress by artificial nutrition and supplementation, such as the supply of diets high in protein and micronutrients. Research has shown that exposure to pesticides alters the gut microbiota, which is necessary for immune function. This emphasizes the necessity of dietary therapies to restore gut health [150]. Although efforts have been made to produce resistant honeybee strains through genetic breeding, studies show that pesticide-induced vulnerabilities cannot be mitigated by more genetic variety alone. When treated to neonicotinoids, even genetically diverse colonies were more vulnerable to parasite infestations [151]. Consequently, regulatory reforms are also crucial. The establishment of more practical safety guidelines requires updated risk assessment models that take into account chronic and sublethal consequences, such as disturbances to immune function, cognition, and circadian rhythms [110]. Finally, a key component of successful mitigation continues to be farmer education and training. A recent systematic analysis highlights the necessity of reevaluating farming methods and giving priority to educating farming communities about pollinator-friendly practices and knowledge [152]. Together, these cutting-edge tactics provide a thorough framework for safeguarding honeybee populations in traditional and non-traditional agricultural systems. Figure 4 illustrates the several key mitigation strategies discussed, providing a visual overview of their role in reducing honeybee exposure to neonicotinoids.

## 8. Conclusions

The sublethal impacts of neonicotinoids on honeybees are much more than the combination of their individual toxicities and they range all the way down to the molecular level and up to the colony level. These outcomes are not similar in all compounds, e.g., imidacloprid and clothianidin have been the most neurotoxic and behavioral deficits, whereas in other cases, thiacloprid has displayed reduced comparative toxicity in some field settings. These differences are indicative of the necessity of taking the individual compounds into account when determining risk, as opposed to simply assuming similarity. Sublethal effects reverberate through an array of biological systems- neuronal dysfunction compromises foraging and navigation, and social organization are disrupted due to changes in pheromonal communication, and immunosuppression increases the susceptibility to parasites such as Nosema ceranae and Varroa destructor. These impairments, when considered alone, are not particularly dramatic but they add and synergies with one another and lead to colony collapse and a reduction in pollination services. Regulating threats of the two most hazardous neonicotinoids needs to be a priority, regulatory policies should phase them out, with the most emphasis being given during flowering seasons when bees are most in danger. The less detrimental chemicals that cannot be discarded must only be incorporated into powerful integrated pest management (IPM) systems. In addition to chemical solutions, landscape-scale measures, including restoring flowering diversity and high-quality pesticide-free forage, are needed to enhance detoxification and resilience of the honeybee population. Additional studies are required to enhance our knowledge of epigenetic alterations, colon activities throughout the lifetime and synergetic stimuli in a natural environmental setting. Such evidence plays a critical role in the process of improving risk thresholds and making differentiated regulatory policies. Saving honeybees is not only a biological requirement but the moral responsibility of man. Their survival requires interdisciplinary approach and the need to think ahead in terms of policy reform regarding changes and global environmental stewardship to optimize biodiversity, food security and agricultural sustainability.

## Data Availability

Not applicable.

## Abbreviations

The following abbreviations are used in this manuscript:

IPM	Integrated Pest Management
nAChRs	Nicotinic Acetylcholine Receptors
EFSA	European Food Safety Authority
CREB	cAMP Response Element-Binding Protein
MAPK	Mitogen-Activated Protein Kinase
VGLUTs	Vesicular Glutamate Transporters
VGCCs	Voltage-Gated Calcium Channels
P450s	Cytochrome P450 Monooxygenases
PPO	Prophenoloxidase
LD50	Lethal Dose 50%
LC50	Lethal Concentration 50%
CV	Conventional Gut Microbiota
GD	Gut Microbiota Deficient
CCD	Colony Collapse Disorder
PI3K	Phosphoinositide 3-Kinase
IAPV	Israeli Acute Paralysis Virus
PER	Proboscis Extension Response
ROS	Reactive Oxygen Species
SOD	Superoxide Dismutase
CAT	Catalase
